# Can public health policies on alcohol and tobacco reduce a cancer epidemic? Australia's experience

**DOI:** 10.1186/s12916-019-1453-z

**Published:** 2019-11-27

**Authors:** Heng Jiang, Michael Livingston, Robin Room, Yong Gan, Dallas English, Richard Chenhall

**Affiliations:** 1 Centre for Alcohol Policy Research, School of Psychology and Public Health, La Trobe University, Level 5, HS2, Bundoora, Melbourne, Victoria 3086 Australia; 2 Centre for Health Equity, Melbourne School of Population and Global Health, The University of Melbourne, Melbourne, Victoria Australia; 3 Centre for Social Research on Alcohol and Drugs, Department of Public Health Sciences, Stockholm University, Stockholm, Sweden; 4 Department of Social Medicine and Health Management, School of Public Health, Tongji Medical College, Huazhong University of Science and Technology, Wuhan, Hubei China; 5 Centre for Epidemiology and Biostatistics, Melbourne School of Population and Global Health, The University of Melbourne, Melbourne, Victoria Australia; 6 Cancer Epidemiology Centre, Cancer Council Victoria, Melbourne, Victoria Australia

**Keywords:** Alcohol, Tobacco, Health policy, Cancer mortality, Time series

## Abstract

**Background:**

Although long-term alcohol and tobacco use have widely been recognised as important risk factors for cancer, the impacts of alcohol and tobacco health policies on cancer mortality have not been examined in previous studies. This study aims to estimate the association of key alcohol and tobacco policy or events in Australia with changes in overall and five specific types of cancer mortality between the 1950s and 2013.

**Methods:**

Annual population-based time-series data between 1911 and 2013 on per capita alcohol and tobacco consumption and head and neck (lip, oral cavity, pharynx, larynx and oesophagus), lung, breast, colorectum and anus, liver and total cancer mortality data from the 1950s to 2013 were collected from the Australian Bureau of Statistics and Cancer Council Victoria, the WHO Cancer Mortality Database and the Australian Institute of Health and Welfare. The policies with significant relations to changes in alcohol and tobacco consumption were identified in an initial model. Intervention dummies with estimated lags were then developed based on these key alcohol and tobacco policies and events and inserted into time-series models to estimate the relation of the particular policy changes with cancer mortality.

**Results:**

Liquor licence liberalisation in the 1960s was significantly associated with increases in the level of population drinking and thereafter of male cancer mortality. The introduction of random breath testing programs in Australia after 1976 was associated with a reduction in population drinking and thereafter in cancer mortality for both men and women. Meanwhile, the release of UK and US public health reports on tobacco in 1962 and 1964 and the ban on cigarette ads on TV and radio in 1976 were found to have been associated with a reduction in Australian tobacco consumption and thereafter a reduction in mortality from all cancer types except liver cancer. Policy changes on alcohol and tobacco during the 1960s–1980s were associated with greater changes for men than for women, particularly for head and neck, lung and colorectum cancer sites.

**Conclusion:**

This study provides evidence that some changes to public health policies in Australia in the twentieth century were related to the changes in the population consumption of alcohol and tobacco, and in subsequent mortality from various cancers over the following 20 years.

## Background

In 2015, cancer was the second leading cause of death globally behind cardiovascular disease [1]. Long-term alcohol and tobacco use are associated with the risk of cancer at a number of body sites [2, 3]. Two systematic reviews on alcohol, tobacco and cancer diseases published by the World Cancer Research Fund (WCRF) in 2007 and the International Agency for Research on Cancer (IARC) in 2012 reported that alcohol use increases the risk of cancers at seven sites: oropharynx, larynx, oesophagus, liver, colon, rectum and female breast, while long-term tobacco smoking is associated with cancers of the lips, oral cavity, pharynx, larynx, lung, stomach, colorectum, breast, pancreas and liver [4, 5]. The dose-response relationships between alcohol, tobacco use and cancer mortality and morbidity have been well-documented in the existing epidemiological studies [6–9]. Substantial research evidence indicates that alcohol and tobacco control policies, such as raising the minimum legal drinking age; introducing random breath testing programs to deter drink-driving, media campaigns and health warning labels on the danger of alcohol and tobacco use; and raising alcohol and tobacco taxes or prices, can reduce alcohol and tobacco consumption considerably [10, 11]. Thus, there is a good reason to believe that population policies for alcohol and tobacco can influence cancer mortality. However, no previous studies have directly estimated the impact of these policies on cancer mortality rates.

**Fig. 1:**
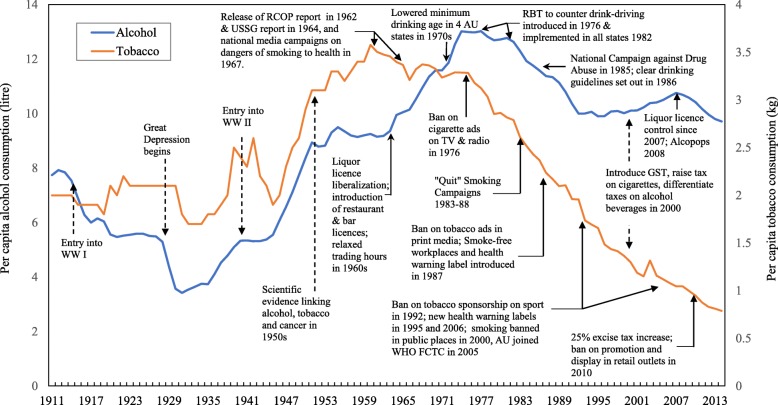
Trends of alcohol and tobacco consumption per capita (15+) in Australia from 1911 to 2013 (solid vertical lines are independent single events potentially affecting alcohol or tobacco, and dotted vertical lines are joint events potentially affecting both alcohol and tobacco consumption) (see detailed summary of key alcohol and tobacco policies and events in Additional file 1)

As in Canada, the UK and the USA [12–15], Australian annual per capita alcohol and tobacco consumption soared after the Second World War (see Fig. 1). Tobacco consumption reached its peak in 1961 and then decreased steadily, with smoking discouraged by a series of tobacco control strategies. While alcohol consumption increased through the 1960s and 1970s, reaching a peak of 13.4 l in 1977, it then decreased to 10 l by 1989 and remained at approximately the same level after that. Such changes have also occurred elsewhere and have attracted a lot of research attention. Many studies nominated particular policy or intervention landmarks [16, 17], such as large government campaigns, advertising bans or increases in the legal drinking age, as the possible cause of changes in consumption trends. Similarly, most studies focused on a single potentially harmful behaviour (i.e. either alcohol or tobacco use) as an outcome. However, the combined effects of a series of public health policy strategies on alcohol and tobacco consumption, and on related cancer mortality, have remained unexamined. The scope of the current study includes policy changes or events which drove the consumption up or down (see Fig. 1). “Policy” is interpreted broadly, to include, for instance, the publication of major official documents, such as the 1964 US Surgeon-General’s report on smoking and health, which attracted global attention.

While some of the policies and events in Fig. 1 happened on a specific date, others such as liquor licence liberalisation and “quit smoking” campaigns happened over a period of time (see Additional file 1: Tables S2 and S3 for the handling of these changes in the analysis). The effects associated with a change in law on a specific date may also occur over a period of time rather than specifically on that date; examples can be found where the effect of a law change came more from the public discourse around the proposed change than from the actual legal change (e.g. Hingson et al. [18] and Møller [19]). In the analyses which follow, we use the effective dates of laws and timing of events in our analyses, but recognise that they are often markers of a wider change in popular understanding and behaviour associated with the event or legal change.

Previous epidemiological studies have suggested that the cumulative effects of long-term alcohol and tobacco consumption on chronic diseases vary by gender [20], because of the differences in drinking and smoking habits between men and women [2, 21]. Our recent studies have confirmed that the associations of population-level alcohol and tobacco consumption with cancer mortality were different for men and women [22], while the relationship was strongest for a model where there were lagged effects of the consumption over a period of 20 years. Furthermore, even with the same amount of long-term alcohol and tobacco consumption, the relative risks for different cancer sites may be different due to the different mechanisms of tumour development in the human body. Thus, we investigate in this paper whether the effects of alcohol and tobacco control policies on cancer mortality are different by sex and by cancer sites.

## Methods

### Overview

This study used a combination of searches of available data and time-series methods to (i) identify the key alcohol and tobacco policy changes in Australia during the period 1940–2018 through reviewing published papers and reports, (ii) investigate the relation of key alcohol and tobacco policies and events to alcohol and tobacco consumption and (iii) examine the relationships between those policies and events, associated with significant changes in alcohol and tobacco consumption and cancer mortality. Only the policies or events which happened in the period from the 1960s to the 1980s were measured in our models. The relatively long lag (e.g. 20 years) between consumption and cancer deaths means that effects of any events happening after the 1980s cannot be captured. Both separate and joint effects of alcohol and tobacco policies on various cancer mortality rates were measured in the analysis.

We searched on Google Scholar and PubMed for journal articles and reports to identify key alcohol and tobacco policy changes in Australia during the period between 1940 and 2018. The search strategy and identified policies and events were summarised in Additional file 1. A Strengthening the Reporting of Observational Studies in Epidemiology (STROBE) statement for the current study is attached in Additional file 2.

### Data

Annual time-series data of head and neck (lip, oral cavity, pharynx, larynx and oesophagus), lung, breast and total cancer mortality from 1950 to 2013 and colorectum and anus cancer mortality from 1955 to 2013 were collected from the WHO Cancer Mortality Database [23], while liver cancer mortality data from 1968 to 2013 were collected from the Australian Institute of Health and Welfare (AIHW) [24]. All the collected cancer data were adjusted by the world age-standardised population [25]. More details of cancer sites and data collection are summarised in Additional file 1.

A proxy for per capita alcohol consumption was constructed, using data on alcohol sales sourced from the Australian Bureau of Statistics (ABS). Data on alcohol consumption per person aged 15+ for the years 1961 to 2013 are taken from a recent synthesis of historical data [26], while data from earlier years (1911–1960) were extracted manually from relevant yearbooks (e.g. Commonwealth Bureau of Census and Statistics report [27]) and converted from gallons or proof gallons to litres of pure alcohol. This was then converted to litres of pure alcohol per resident aged 15 and older, using population data provided by the AIHW [24]. Data on per capita tobacco consumption (aged 15+) from 1911 to 2013 were collected from Cancer Council Victoria [28] and a 2016 KPMG report, *Illicit Tobacco in Australia* [29]. Male breast cancer cases were very few or none in a number of years in the study period; thus, we excluded male breast cancer mortality in the analysis.

### Statistical model

Autoregressive integrated moving average (ARIMA) models with intervention dummies were employed in this study. When a time series has a unit root, the series is non-stationary and the ordinary least squares estimator is not normally distributed and would lead to a spurious regression [30]. The augmented Dickey-Fuller (ADF) unit root test is commonly used for testing for stationarity [31]. Furthermore, the error term (which includes explanatory variables not considered in the model) is allowed to have a temporal structure that is modelled and estimated in terms of autoregressive or moving average parameters [32]. In most cases, a differencing of the time series is sufficient to eliminate non-stationarity [33]. The ARIMA model with dummy variables can be written as follows:

$$ {\Delta  Y}_t=\alpha +{\beta \Delta  Y}_{t-1}+\mu {\Delta  C}_{i,t}+{\sum}_j^n{\gamma}_j{D}_{j,t}+{\delta \Delta  E}_{t-1} $$

where *∆* is the differencing operator, *Y*_*t*_ represents the dependent variable at time *t* (sex-specific cancer mortality rates—number of deaths per 100,000 population), *α* is a constant (which marks the average annual changes due to other causes), *β* is the coefficient value of the AR (1) term, *C*_*i*, *t*_ is the control variables considered in the estimation, *i* is the number of control variables, *μ* is the coefficient values of the control variables, *δ* is the coefficient value of the MA (1) term (*∆E*_*t* − 1_), *D*_*j*, *t*_ is the one-off event dummy variable *j* at time *t*, *n* is the number of dummy variables and *γ*_*j*_ is the estimates of the effect of the events or interventions. One-off event dummy variables have been widely applied in many previous time-series studies to analyse the effect of seasonality, major changes in policy and financial crises [34–37]. The intervention remains at value 1 for the duration of the presence of the event; otherwise, the intervention is 0 during the period of the absence of the event (e.g. a dummy variable was coded as 1 between 1984 and 1988 for the event of “State Quit Smoking Campaigns in 1984–1988” and coded as 0 for other years).

In order to estimate better the effects of key state-level alcohol and tobacco policies, the key state-level policy dummies were constructed based on a roll-out approach, adding in the population weight of each state when the health policy was introduced in that state. For example, the dummy variable for the random breath testing (RBT) program was coded as 0.25 in 1976, as RBT was first introduced in Victoria (with 25% of the national population) in 1976. After that, the RBT dummy variable was recoded, adding the population weight of each state when RBT was introduced in that state. The RBT dummy variable was finally coded as 1 after 1987, as it was fully introduced in all states (the development of dummy variables for key national- and state-level alcohol and tobacco policies or events is elaborated in Additional file 1: Tables S2 and S3).

When estimating the effects of policies on male cancer mortality, female cancer mortality data were used as a control variable to control for other aetiological factors (e.g. improvements in treatment, nutrition [38, 39]) which may affect male cancer mortality (this approach has been used in previous studies on all-cause mortality [33, 40]). Men were the predominant consumers of alcohol and tobacco in Australia in the time period studied; in the 1940s, the smoking prevalence was 26% for females and 72% for males [28], while for alcohol consumption in the same period, men drank around 1.6 standard drinks per day compared with 0.2 standard drinks per day for women [41]. Changes in the per capita consumption will therefore have a larger impact on men’s than on women’s health. Thus, a model that controlled for male cancer mortality would substantially underestimate the effects of alcohol and tobacco use on women’s cancer mortality, so no control variable was used in female cancer mortality models.

The model fit was evaluated with the aid of the Box-Ljung portmanteau test of the first ten autocorrelations, Q (10) (i.e. residuals without serial correlation). All analyses were completed by EViews version 10 software.

It is worth noting that the changes in the ICD code for cancer diseases in the last 60 years may have some impact on the cancer death records in the death registry in Australia. Dummy variables were included in initial models to assess the impact of changes in coding practices, but there were no significant effects on cancer mortality rates, so they were excluded from the final models. Further changes in coding practices over time could not be adjusted for, but may have also influenced recorded mortality rates.

### Lags between policy change and mortality rates

**Fig. 2:**
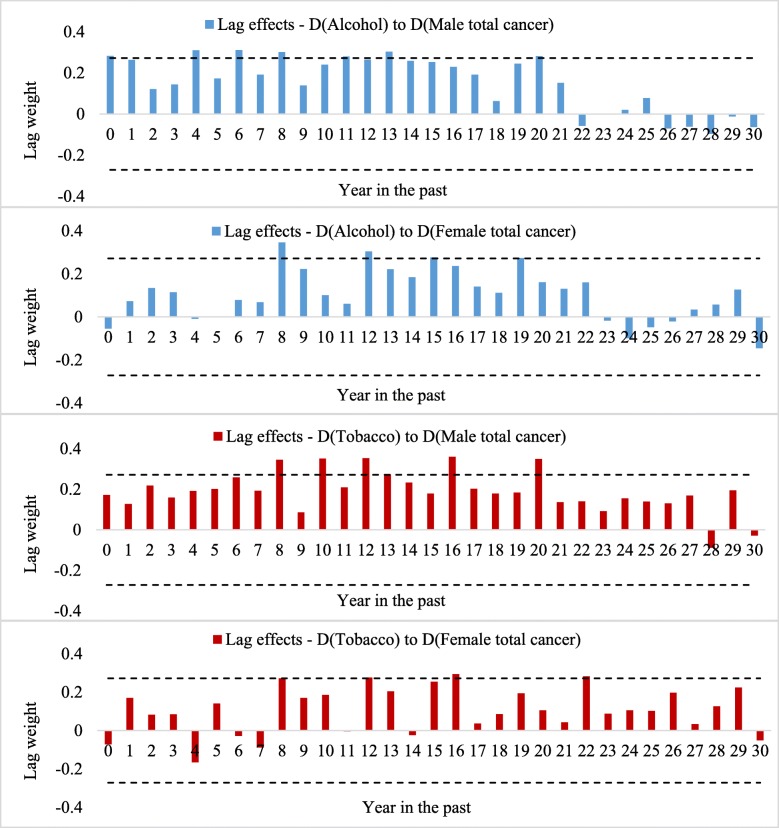
Lag length of changes in population drinking and smoking on male and female mortality rates of overall cancer. (The critical values of the cross-correlation test were calculated based on |± 2/$\sqrt{n-10}$| = 0.272, and *n* = 64 is number of years in the sample)

Any effects on cancer mortality of the changes in population drinking and smoking will not be fully seen in the year in which the change occurs. Following the methods used in our previous studies [22, 42, 43], we conducted cross-correlation tests to explore the cross-correlation relationships between per capita alcohol and tobacco consumption and rates of male and female total cancer mortality in Australia to identify lag lengths of long-term alcohol and tobacco use on cancer mortality. The results of a cross-correlation test (based on the first differenced data with trends and autocorrelation removed) between alcohol and tobacco consumption and male and female cancer mortality are presented in Fig. 2.

A significant and positive correlation was found between alcohol consumption and male and female cancer mortality, and the results suggest that 20- and 19-year lags on alcohol consumption could be applied in the male and female cancer estimation models, respectively. The cross-correlation analyses also indicate that lag lengths for the associations between smoking and male and female cancer mortality are 20 and 22 years, respectively.

The policies associated with significant changes in alcohol and tobacco consumption were first identified in the initial estimation. These policies or events were then related to consumption and subsequently to cancer mortality. The estimated lag lengths between alcohol and tobacco consumption and cancer mortality were applied to the developed policy or intervention dummies. Furthermore, the lagged dummies of the identified key alcohol and tobacco policies or events were inserted into the time-series models to estimate the effects of these events on cancer mortality rates in Australia. The lagged effects of alcohol and tobacco consumption on cancer mortality could be up to 20 or 22 years, and our lagging models take account of the wide time spread of the effects.

## Results

The ADF unit root test is used to test the stationarity of the time series in this study (Additional file 1: Table S4). The unit root test results suggest that alcohol consumption, tobacco consumption and sex-specific different types of cancer mortality are non-stationary in data at the untransformed level and become stationary after the first differencing at the significance level of 0.05.

### The relationship between alcohol and tobacco policies and consumption levels

**Fig. 3:**
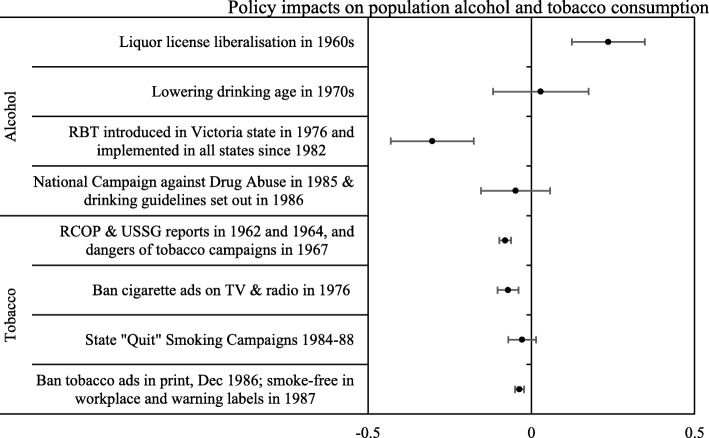
The effects of key alcohol and tobacco public health policies during the 1960s and 1980s on population drinking and smoking in Australia (95% CI bars added)

The relationship of key alcohol and tobacco public health policies during the period 1960s–1980s with changes in population drinking and smoking in Australia is presented in Fig. 3 (coefficient values and 95% CIs of the initial ARIMA model that estimated the impact of policies or events on alcohol and tobacco consumption are presented in Additional file 1: Table S5). It shows that liquor licence liberalisation in the 1960s, a policy change reflecting and enabling a widening acceptance of alcohol in Australian homes and public life [44], was associated with a significant increase in alcohol consumption in Australia [Coef. = 0.236 (95% CI 0.124, 0.348)]. In contrast, a significant decrease in alcohol consumption was found to be associated with the introduction of random breath testing (RBT) programs to discourage and penalise drink-driving [− 0.303 (− 0.43, − 0.176)]. Release of the Royal College of Physicians (RCP) and US Surgeon General (USSG) reports in 1962 and 1964, and national health media campaigns about the dangers of tobacco in 1967, was found to be associated with significant reductions in tobacco consumption [− 0.08 (− 0.098, − 0.062)]. A decreasing trend in tobacco consumption was also found in the wake of banning cigarette ads on TV and radio in 1976 [− 0.071 (− 0.103, − 0.039)], banning tobacco ads in print media in December 1986 and introducing smoke-free rules at the workplace and health warning labels in 1987 [− 0.036 (− 0.050, − 0.022)]. No significant relations with changes in alcohol consumption were found for lowering the minimum drinking age in the 1970s, the National Campaign against Drug Abuse in 1985 and setting out drinking guidelines in 1986, or in tobacco consumption from the state “Quit” Smoking Campaigns in 1984–1988. The policies which thus appeared to be associated with significant changes in alcohol or tobacco consumption were identified as the key influencing policies. The estimated lag lengths were applied to these key influencing policies to estimate the effects of those policies on cancer mortality.

### The relationship between key alcohol and tobacco policy changes and cancer mortality

**Fig. 4:**
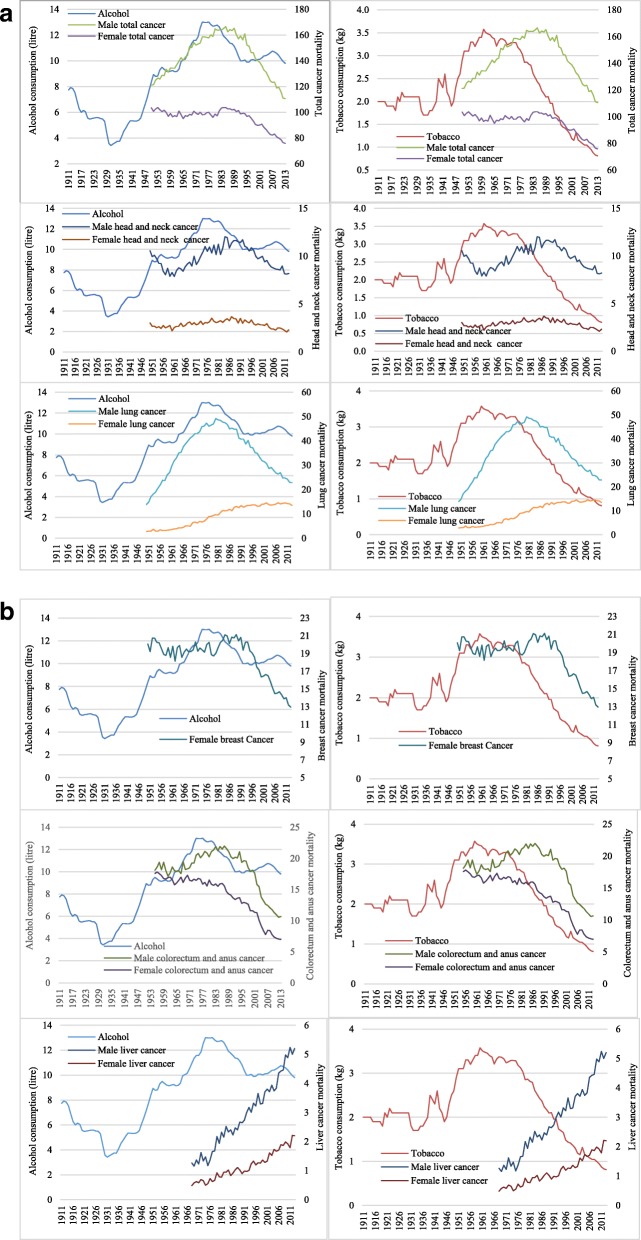
Alcohol and tobacco consumption per capita (15+) and total cancer, head and neck (lip, oral cavity, pharynx, larynx and oesophagus) cancer, lung cancer (**a**), colorectum and anus cancer, liver cancer mortality rates among male and female, and female breast cancer (**b**) mortality per 100,000 population. Head and neck, lung, breast and total cancer mortality data from 1950 to 2013 and colorectum and anus cancer mortality from 1955 to 2013, were collected from the WHO Cancer Mortality Database, while liver cancer mortality (from 1968 to 2013) data were collected from the Australian Institute of Health Welfare; all the collected cancer data were adjusted by the world age-standardized population (Segi 1960) [25]

Trends of alcohol and tobacco consumption per capita between 1911 and 2013 and trends of the overall and five specific types of cancer mortality between the 1950s and 2013 are shown in Fig. 4. Trends of male and female overall and different cancer type mortality had similar patterns, with 15–20 years lagged movements observed following the changes in alcohol and tobacco consumption, except for liver cancer mortality rates, which have increased sharply in the last 50 years.

Three models were developed to analyse the association of these key alcohol and tobacco policies with lagged cancer mortality, including two models of separate relationships of alcohol and tobacco policies and one overarching model of the joint relationships of key alcohol and tobacco policies. The estimated results are summarised in Tables 1 and 2. They show that liquor licence liberalisation in the 1960s was associated with significant increases in Australian mortality from head and neck [0.11 (0.03, 0.20)], lung [1.80 (1.37, 2.23)], colorectum [0.58 (0.31, 0.84)] and overall cancers [2.13 (1.96, 2.30)] among men, and head and neck [0.04 (0.02 to 0.06)] and colorectum cancers [0.18 (0.09, 0.27)] among women. In contrast, the introduction of RBT programs starting in 1976 and the ban on cigarette ads on TV and radio in 1976 were associated with protective impacts on overall cancer mortality among both men [− 1.01 (− 1.37, − 0.64) and − 1.43 (− 2.31, − 0.54)] and women [− 1.25 (− 2.01, − 0.49) and − 1.24 (− 2.34, − 0.14)]. These results reveal that by 20 years after the introduction of RBT programs and the ban on cigarette ads on TV and radio, the annual overall cancer mortality rates were reduced by 1.01 and 1.43 per 100,000 population for men and 1.25 and 1.24 per 100,000 population for women, respectively. The release of the RCP report in 1962, the USSG report in 1964 and health media campaigns about the dangers of tobacco in 1967 were found to be associated with significant reductions in head and neck [− 0.10 (− 0.16, − 0.05)], lung [− 1.31 (− 1.86, − 0.76)] and overall cancers [− 2.18 (− 2.76, − 1.60)] in men and only in head and neck cancer [− 0.04 (− 0.06, − 0.01)] in women. The associations of alcohol and tobacco policies or events with male and female liver cancer mortality were statistically insignificant.

**Table 1:** The separate and joint relations of key alcohol and tobacco public health policies to men’s cancer mortality (deaths per 100,000 population) in Australia

Models	Men^c^				
	Head and neck cancer, Coef. (95% CI)	Lung cancer, Coef. (95% CI)	Colorectum cancer, Coef. (95% CI)	Liver cancer, Coef. (95% CI)	All cancer sites, Coef. (95% CI)
Separate relation of alcohol and tobacco policies to cancer mortality					
Alcohol^a^					
Liquor licence liberalisation in the 1960s	0.11 (0.03 to 0.20)*	1.80 (1.37 to 2.23)***	0.58 (0.31 to 0.84)***	− 0.00 (− 0.10 to 0.10)	2.13 (1.96 to 2.30)***
RBT introduced in Victoria in 1976 and fully implemented in all states in 1982	− 0.14 (− 0.23 to − 0.78)**	− 0.28 (− 0.77 to 0.22)	− 0.86 (− 1.43 to − 0.29)***	− 0.04 (− 0.13 to 0.05)	− 1.01 (− 1.37 to − 0.64)***
Tobacco^b^					
Release of RCOP and USSG reports in 1962 and 1964 and health media campaigns about the dangers of tobacco in 1967	− 0.10 (− 0.16 to − 0.05)***	− 1.31 (− 1.86 to − 0.76)***	− 0.15 (− 0.35 to 0.06)	− 0.00 (− 0.09 to 0.08)	− 2.18 (− 2.76 to − 1.60)***
Cigarette ads banned on TV and radio in 1976	− 0.21 (− 0.31 to − 0.12)***	− 0.85 (− 1.41 to − 0.30)**	− 0.55 (− 0.82 to − 0.28)***	− 0.00 (− 0.09 to 0.09)	− 1.43 (− 2.31 to − 0.54)**
Tobacco ads banned in print media in December 1986 and smoke-free workplace rules and health warning label introduced in 1987	− 0.14 (− 0.24 to − 0.05)**	− 0.24 (− 0.70 to 0.21)	− 0.25 (− 0.58 to 0.08)	0.09 (− 0.01 to 0.20)	− 0.43 (− 1.47 to 0.61)
Joint relation of alcohol and tobacco policies to cancer mortality^a,b^					
Liquor licence liberalisation in the 1960s	0.09 (− 0.22 to 0.40)	1.04 (− 0.41 to 2.48)	0.08 (− 0.41 to 0.57)	− 0.09 (− 0.24 to 0.06)	0.80 (0.02 to 1.59)*
RBT introduced in Victoria in 1976 and fully implemented in all states in 1982	− 1.43 (− 1.96 to − 0.90)***	− 0.21 (− 0.64 to 0.22)	− 1.21 (− 2.31 to − 0.10)**	− 0.26 (− 0.47 to − 0.06)*	− 1.31 (− 1.70 to − 0.92)***
Release of RCOP and USSG reports in 1962 and 1964 and health media campaigns about the dangers of tobacco in 1967	− 0.70 (− 1.09 to − 0.31)**	− 1.95 (− 2.76 to − 1.13)***	− 0.19 (− 1.05 to 0.82)	− 0.28 (− 0.47 to − 0.09)**	− 4.27 (− 5.90 to − 2.64)***
Cigarette ads banned on TV and radio in 1976	− 0.31 (− 0.44 to − 0.19)***	− 1.21 (− 2.06 to − 0.37)**	− 0.78 (− 1.04 to − 0.53)***	− 0.04 (− 0.09 to 0.00)	− 2.20 (− 3.17 to − 1.24)***
Tobacco ads banned in print media in December 1986 and smoke-free workplace rules and health warning labels introduced in 1987	0.08 (− 0.22 to 0.39)	− 0.39 (− 0.96 to 0.18)	− 0.15 (− 0.83 to 0.53)	0.16 (− 0.09 to 0.41)	− 0.97 (− 1.50 to − 0.45)***

*p < 0.05, **p < 0.01, ***p < 0.001. The unit of the coefficients is the number of deaths per 100,000 population, and the coefficient values can be interpreted as the number of cancer deaths per 100,000 population reduced or increased annually in an association with the implementation of alcohol and tobacco policies in the 20 years before. ^a^Lag lengths of alcohol policies on male and female cancer mortality are 20 and 19 years, respectively. ^b^Lag lengths of tobacco policies on male and female cancer mortality are 20 and 22 years, respectively (lag length estimation was shown in Fig. 1). RBT is a random breath testing program. Head and neck cancer includes lip, oral cavity, pharynx, larynx and oesophagus cancers. ^c^Female cancer mortality was used as a control variable in the models

**Table 2:** The separate and joint relations of key alcohol and tobacco public health policies to cancer mortality (deaths per 100,000 population) in Australian women

Models	Women					
	Head and neck cancer, Coef. (95% CI)	Lung cancer, Coef. (95% CI)	Breast cancer, Coef. (95% CI)	Colorectum cancer, Coef. (95% CI)	Liver cancer, Coef. (95% CI)	All cancer sites, Coef. (95% CI)
Separate relation of alcohol and tobacco policies to cancer mortality						
Alcohol^a^						
Liquor licence liberalisation in the 1960s	0.04 (0.02 to 0.06)***	0.17 (− 0.10 to 0.44)	− 0.05 (− 0.25 to 0.15)	0.18 (0.09 to 0.27)***	0.01 (− 0.09 to 0.11)	0.22 (− 0.47 to 0.90)
RBT introduced in Victoria in 1976 and fully implemented in all states in 1982	− 0.06 (− 0.08 to − 0.03)***	− 0.23 (− 0.47 to 0.01)	− 0.40 (− 0.62 to − 0.17)**	− 0.18 (− 0.27 to − 0.09)***	0.03 (− 0.06 to 0.11)	− 1.25 (− 2.01 to − 0.49)**
Tobacco^b^						
Release of RCOP and USSG reports in 1962 and 1964 and health media campaigns about the dangers of tobacco in 1967	− 0.04 (− 0.06 to − 0.01)**	− 0.03 (− 0.19 to 0.12)	− 0.05 (− 0.23 to 0.14)	− 0.15 (− 0.31 to 0.01)	0.01 (− 0.08 to 0.12)	0.05 (− 0.80 to 0.90)
Cigarette ads banned on TV and radio in 1976	− 0.07 (− 0.11 to − 0.02)**	− 0.23 (− 0.43 to 0.03)*	− 0.44 (− 0.68 to − 0.20)***	− 0.26 (− 0.47 to − 0.05)*	0.00 (− 0.10 to 0.10)	− 1.24 (− 2.34 to − 0.14)*
Tobacco ads banned in print media in December 1986 and smoke-free workplace rules and health warning label introduced in 1987	0.04 (− 0.03 to 0.10)	− 0.04 (− 0.28 to 0.20)	0.02 (− 0.27 to 0.31)	0.24 (− 0.01 to 0.50)	0.02 (− 0.09 to 0.14)	0.19 (− 1.09 to 1.46)
Joint relation of alcohol and tobacco policies to cancer mortality^a,b^						
Liquor licence liberalisation in the 1960s	0.04 (− 0.10 to 0.17)	0.05 (− 0.48 to 0.58)	1.19 (0.79 to 1.59)***	0.41 (− 0.10 to 0.97)	− 0.03 (− 0.10 to 0.05)	1.11 (− 0.53 to 2.75)
RBT introduced in Victoria in 1976 and fully implemented in all states in 1982	− 0.41 (− 0.66 to − 0.15)**	0.19 (− 0.06 to 0.45)	− 0.99(− 1.69 to − 0.28)**	− 0.91 (− 1.74 to − 0.08)*	0.06 (− 0.05 to 0.17)	− 0.69 (− 1.09 to − 0.28)**
Release of RCOP and USSG reports in 1962 and 1964 and health media campaigns about the dangers of tobacco in 1967	− 0.25 (− 0.46 to − 0.04)*	− 0.42 (− 0.83 to − 0.01)*	− 0.369(− 0.98 to 0.20)	− 0.15 (− 0.86 to 0.56)	− 0.07 (− 0.18 to 0.04)	− 3.91 (− 5.74 to − 2.09)***
Cigarette ads banned on TV and radio in 1976	− 0.08 (− 0.57 to 0.40)	− 0.37 (− 0.49 to − 0.25)***	− 0.25 (− 0.44 to − 0.06)**	− 0.06 (− 0.31 to 0.18)	0.02 (0.00 to 0.05)	− 1.76 (− 2.79 to − 0.72)**
Tobacco ads banned in print media in December 1986 and smoke-free workplace rules and health warning labels introduced in 1987	− 0.01 (− 0.07 to 0.06)	− 0.13 (− 0.27 to 0.01)	− 0.21 (− 0.42 to − 0.01)*	0.05 (− 0.33 to 0.42)	0.01 (− 0.03 to 0.05)	0.20 (− 0.50 to 0.90)

*p < 0.05, **p < 0.01, ***p < 0.001. The unit of the coefficients is the number of deaths per 100,000 population, and the coefficient values can be interpreted as the number of cancer deaths per 100,000 population reduced or increased annually in an association with the implementation of alcohol and tobacco policies in the 20 years before. ^a^Lag lengths of alcohol policies on male and female cancer mortality are 20 and 19 years, respectively. ^b^Lag lengths of tobacco policies on male and female cancer mortality are 20 and 22 years, respectively (lag length estimation was shown in Fig. 1). RBT is a random breath testing program. Head and neck cancer includes lip, oral cavity, pharynx, larynx and oesophagus cancers

Similar relationships were found in the joint effect model, indicating that the five identified key alcohol and tobacco policy strategies were significantly associated with overall cancer mortality for men and three policy strategies had significant relationships with overall cancer mortality for women.

## Discussion

This is the first study to evaluate the association of public health policies on alcohol and tobacco with cancer mortality. The study provides evidence that two alcohol policies and three tobacco policies implemented during the 1960s and 1980s, along with the normative shifts they reflected and facilitated, were associated with significant effects on population drinking and tobacco smoking, which were accompanied by significant changes in mortality from various cancers around 20 years later.

The results of the joint effect models suggest that the increases in alcohol licences in the 1960s, reflecting and promoting shifts in alcohol’s cultural position, were associated with increases in overall cancer mortality for men and breast cancer mortality for women in Australia. In contrast, for alcohol the introduction of the RBT program, and for tobacco the release of the RCP and USSG reports in 1962 and 1964, health media campaigns about the dangers of tobacco in 1967, and the ban on cigarette ads on TV and radio in 1976, were associated with important reductions in cancer mortality among both men and women. Weaker relationships were found from banning tobacco ads in print media in December 1986, and introducing smoke-free workplace rules and health warning labels in 1987, for male overall cancer mortality only. Our study findings suggest that a series of alcohol and tobacco control policies were associated with changes in cancer mortality rates which were gradual, persistent and complementary to each other in the long term, although for alcohol, the result was a reversal in trends between the earlier and later parts of the postwar period.

Both male and female liver cancer mortalities in Australia have increased dramatically in the last 60 years, not obviously reflecting changes in alcohol and tobacco consumption per capita. Female liver cancer mortality was not associated with alcohol and tobacco policies in either separate or joint effects models. For alcohol, introducing the RBT program beginning in 1976, and for tobacco the release of the RCP and USSG reports in 1962 and 1964, and health media campaigns about the dangers of tobacco in 1967, were associated with small reductions in male liver cancer mortality. The weak relationship between the trends in alcohol consumption and liver cancer mortality could be due to the dramatic increase in the prevalence of HCV, HBV and diabetes since the 1960s [45, 46]—the major risk factors for liver cancer diseases in Australia [47]. The long-term prevalence rates of HBV, HCV and diabetes are not available for Australia, so these confounding effects cannot be controlled for, meaning that the relationships of alcohol and tobacco policies with liver cancer mortality could be considerably underestimated.

The results also suggest that there was no statistically significant association between some alcohol and tobacco policies or events and eventual cancer mortality. These included lowering the minimum legal drinking age in the 1970s, the National Campaign against Drug Abuse in 1985 and the issuance of drinking guidelines in 1986, and state “Quit” Smoking Campaigns in 1984–1988. These policies may have had a variety of other impacts: we have shown, for example, that drinking age policies reduced traffic deaths [37]; but this may have been by such means as changing the location rather than the amount of drinking. Changing the price or tax levels has been considered as an important policy on alcohol and tobacco consumption in many countries [48, 49]. But in Australia, key changes on alcohol and tobacco prices and taxes occurred only after 1990, while during the 1950s–1980s period, alcohol and tobacco prices were only adjusted according to the inflation rate, so this factor had little confounding effect in our models.

The correlations of alcohol and tobacco consumption with male and female cancer mortality are different, with lag lengths varying from 19 to 22 years. Moreover, the variations in the associations of different alcohol and tobacco control policies with different cancer mortality among men and women indicate that policy changes on alcohol and tobacco during the 1960s–1980s period were associated with greater effects on men than on women, particularly for upper aerodigestive tract, lung and colorectum cancer sites. Heavy drinking and smoking were more common and heavier among men than among women, so stronger relationship for men probably reflects that the risk curves for many cancers are curvilinear, rising more rapidly at higher consumption levels [50]. The release of the RCP and USSG reports in 1962 and 1964, and health media campaigns about the dangers of tobacco in 1967—official policies which fed into changing the cultural position of tobacco—was found to be associated with greater effects than other tobacco control policies in the study period.

In the head and neck cancer estimation, the introduction of RBT program since 1976 and the release of the RCP and USSG reports in 1962 and 1964 and health media campaigns about the dangers of tobacco in 1967 were associated with reductions in both men and women mortality rates. While alcohol policy changes were not related to lung cancer mortality in the joint model, reductions in both men and women lung cancer mortalities were significantly associated with the release of the RCP and USSG reports in 1962 and 1964 and with health media campaigns about the dangers of tobacco in 1967 and the ban on cigarette ads on TV and radio in 1976. Four out of five key alcohol and tobacco policies were found to be significantly related to delayed reductions in the female breast cancer mortality rate. Although the relationship between alcohol and female breast cancer had been widely documented, the link between smoking and female breast cancer remains controversial [51, 52]. Nevertheless, a recent meta-analysis has suggested a positive association between smoking and breast cancer mortality [53]. Our study results have shown a negative association between tobacco policy changes and breast cancer mortality, indicating that the tobacco ads banned in print media in December 1986 and smoke-free workplace rules and health warning labels introduced in 1987 were followed a considerable time later by falls in female breast cancer mortality in Australia.. Risk factors pushing in the opposite direction in this period may include changes in body mass index and use of exogenous hormones [54, 55]. However, long-term time-series data for these factors were unavailable in Australia. Since male breast cancer cases were very few and unable to be measured in our model, we investigated female breast cancer mortality only.

We found some associations that seem inconsistent with the broader literature on tobacco and cancer [56, 57]. For example, we found that restrictions on tobacco advertising on television were associated with declines in breast cancer mortality but not lung cancer. But while tobacco is a possible risk factor for breast cancer, the attributable fractions are much higher for lung cancer, making these inconsistent relationships implausible. Further, given the existing evidence that the relative risk for breast cancer mortality for smoking is around 1.1 [53], the magnitude of the effects found for tobacco policies in our model is improbably high. These inconsistencies are likely due to other confounding factors (especially changes in screening and treatment [58]) as well as uncertainties in the lag structures modelled and imprecision in the policy impacts, given the range and variety of policies modelled here. Thus, the broader results of our study, while suggestive of substantial links between policy and cancer deaths, should be treated cautiously.

There are some policy implications of this study. First, the results support the proposition that key public health policies that control alcohol and tobacco consumption are effective in reducing cancer mortality in the long term. These effects do not occur in a vacuum: “often, it is the discourse and debate about new legislation which produces an initial effect, more than the law itself [18]. In democratic societies, adoption of or changes in legal and other formal social controls tend to follow popular sentiment. They institutionalise and crystallise what has already been happening at the level of informal social change” [59], and often extend its reach. We recognise, also, that formal policy changes often “go hand-in-hand with informal social changes” in popular discourse and sentiment, “with the causal arrows pointing in both directions” [59]. Second, while for tobacco in the 1960s–1980s the main policy changes associated with significant effects were in terms of health campaigns and advertising restrictions (Fig. 4), for alcohol, the most significant relationships were from newly effective enforcement of drink-driving restrictions in one direction, and increased market availability of alcohol in the other.

As discussed earlier, other confounding factors which may affect cancer mortality of men and women were not considered in our models. Cultural, economic and public opinion factors may affect drinking and smoking behaviours, and, as we have noted, effects attributed to policy changes may also reflect that changes in such factors may have made the policy changes possible and have contributed to the effects. Cumulative drinking and smoking histories were not available, and these variables were not included in our estimations. There has been a marked improvement in the medical treatment of cancer diseases in the last 60 years, which had a substantial impact on cancer mortality (i.e. living longer with cancer and going into remission). Furthermore, we were unable to evaluate in our models whether there was some impact from local public health events on alcohol and tobacco control. Nevertheless, these impacts were partially controlled in our models by using female cancer mortality as a control variable when we estimated male cancer mortality. While no control variable was used in the female cancer mortality estimation, the use of first-differenced data over as long as a period over 60 years and of controls for serial correlation can partially control for trends in unmeasured confounders, and factors that cannot be measured in our models are also likely to be captured by the model residuals. It is worth noting that our study found limited or null effects on mortality for some policy interventions with consistent evaluation evidence of effectiveness at reducing consumption. Our aim in this study is to estimate the aggregate-level associations between policy changes and mortality outcomes, but these relationships are potentially confounded by a variety of other changes in risk behaviours as well as shifts in screening and treatment practices. Combined with the uncertain lags, our findings should thus be treated with a degree of caution. Future work will use long-term cohort data to validate and expand upon these aggregate findings.

## Conclusions

Whatever its limitations, this study has provided new evidence that key public health policies implemented during the 1960s and 1980s that contributed to the changes in population-level drinking and tobacco smoking are related to the changes in mortality rates for various cancers over the subsequent 20-year period. The findings underline that the effects of changes in alcohol and tobacco policies cannot be fully evaluated, for instance, with a 6-month or even a 2-year follow-up [60, 61] and that the effects of policies affecting today’s smoking or drinking behaviour can extend over decades. The modelling approach we have developed here can be used in other countries to evaluate the impact of historic alcohol and tobacco control policies on cancer mortality.

## Supplementary information

**Additional file 1.** Search strategy. Summary of key public health policies on alcohol and tobacco and other intervention events in Australia between 1911 and 2013.. Description of cancer mortality data (ICD-10) and description of cancer mortality data used in the study were summarized in the file. Development of dummy variables for key public health policies or events. Test for stationarity. Estimates of ARIMA models on the relationships of alcohol and tobacco policy with alcohol and tobacco consumption. Alcohol and tobacco consumption (Age 15+) and skin cancer mortality in Australia.

**Additional file 2.** STROBE checklist.

## Data Availability

All data generated or analysed during this study are included in this published article and its supplementary information files.

## Abbreviations

ABS: Australian Bureau of Statistics
ADF: Augmented Dickey-Fuller
AIHW: Australian Institute of Health and Welfare
ARIMA: Autoregressive integrated moving average
HBV: Hepatitis B virus
HCV: Hepatitis C virus
IARC: International Agency for Research on Cancer
RBT: Random breath testing
RCOP: Royal College of Physicians
USSG: US Surgeon General
WCRF: World Cancer Research Fund
WHO: World Health Organization
