# Supplementary material for: Can public health policies on alcohol and tobacco reduce a cancer epidemic? Australia's experience
Source: BMC Med. 2019 Nov 27;17:213. doi: 10.1186/s12916-019-1453-z (PMC6880568; doi:10.1186/s12916-019-1453-z)
Supplement: Supplementary file 1 — Additional file 1. Search strategy. Summary of key public health policies on alcohol and tobacco and other intervention events in Australia between 1911 and 2013.. Description of cancer mortality data (ICD-10) and description of cancer mortality data used in the study were summarized in the file. Development of dummy variables for key public health policies or events. Test for stationarity. Estimates of ARIMA models on the relationships of alcohol and tobacco policy with alcohol and tobacco consumption. Alcohol and tobacco consumption (Age 15+) and skin cancer mortality in Australia. [file 12916_2019_1453_MOESM1_ESM.docx]

**Additional file 1**

**1.1 Search strategy**

We searched PubMed and Google Scholar for journal articles, reviews and reports from 1^st^ Jan 1940 to 31^st^ Oct 2018 to identify studies evaluate national or state level alcohol and tobacc policies or events during 1940 and 2013 in Australia. We used ‘smoking’ or ‘cigarette’ or ‘tobacco’ or ‘alcohol’ or “drinking” or “ethanol” combined with ‘policy’ or ‘event’ and “Australia” as search terms.

The full search terms were presented as below:

((((("ethanol"[MeSH Terms] OR "ethanol"[All Fields] OR "alcohol"[All Fields] OR "alcohols"[MeSH Terms] OR "alcohols"[All Fields]) OR ("tobacco"[MeSH Terms] OR "tobacco"[All Fields] OR "tobacco products"[MeSH Terms] OR ("tobacco"[All Fields] AND "products"[All Fields]) OR "tobacco products"[All Fields])) OR ("tobacco products"[MeSH Terms] OR ("tobacco"[All Fields] AND "products"[All Fields]) OR "tobacco products"[All Fields] OR "cigarette"[All Fields])) OR ("drinking"[MeSH Terms] OR "drinking"[All Fields] OR "alcohol drinking"[MeSH Terms] OR ("alcohol"[All Fields] AND "drinking"[All Fields]) OR "alcohol drinking"[All Fields])) OR ("smoking"[MeSH Terms] OR "smoking"[All Fields])) AND (("policy"[MeSH Terms] OR "policy"[All Fields]) OR event[All Fields]) AND ("australia"[MeSH Terms] OR "australia"[All Fields]) AND ("1940/01/01"[PDAT] : "2018/10/31"[PDAT])

The initial search returned 2007 articles or reports. With duplicate and irrelevant studies (papers/reports were not accessible, no national or state alcohol or tobacco policies mentioned, policies or events not in Australia, or policies or events not between 1940s and 2013 etc.) removed, 44 articles remained. We further identified 3 studies from reference checks meaning 47 articles were included in the review to identify key alcohol and tobacco policies in the study period.

The key public health policies on alcohol and tobacco or other intervention events were identified as the policies or events mentioned in previous studies that have had or may potentially have had impacts on alcohol or tobacco consumption or alcohol and tobacco related harms. Key alcohol and tobacco policies were selected based on the following criteria, in order:

1. The policy/event was mentioned in published government reports or other documents. If there were duplicate studies, we referred to the most recent published articles or reports.
2. The policy/event was evaluated in terms of its impact on consumption or related harms.
3. The policy/event impact was not evaluated. However, it may have had potential to drive the alcohol or tobacco consumption/harm change. This was based on plausibility of any effect and the authors’ observation of historical time series data.

We extracted data on: the year of the policy or event, a description of the policy or event, the effects of the policy or event if estimated and studyauthors. Discrepancies were resolved by discussion and referencing the original publication. The detailed outcomes (reduced injuries, decrease alcohol or tobacco consumption) of the policies or events were not collected and this was beyond the study scope.

**1.2 Summary of key public health policies on alcohol and tobacco and other intervention events in Australia between 1911 and 2013**

***Key joint policies/events between 1911 and 2013***

- 1914-1918, World War I [1, 2]
- 1929-1941, The Great Depression [3, 4].
- 1939-1945, World War II [1, 4, 5].
- 1940s-50s, A series of major medical reports confirmed that alcohol and tobacco use caused a range of serious diseases, including cancer [6-8].
- 2000, The introduction of Goods and Services Tax in Australa led to a tax increase on cigarattes and differentiated taxes on alcohol beverages [9, 10].

***Key tobacco policies/events after 1950s***

- 1962, release of the Royal College of Physicians (RCOP) report on smoking in relation to cancer of the lung and other diseases [11].
- 1964, release of United States Surgeon General (USSG) report indicated that that smoking is "the single greatest cause of avoidable morbidity and mortality in the United States” [12].
- the Cancer Council and the Health Department undertook public media campaigns about the dangers of tobacco in 1967 [1].
- 1976, a national ban on tobacco & cigarette advertisements on TV & radio commenced in September [13].
- 1983-88, Statewide "Quit" Smoking Campaigns [1, 14].
- 1987, tobacco advertising was banned in all print media in December 1986 [15, 16] and under the *Tobacco Act 1987* in Victoria and then other states, smoking is prohibited in all enclosed workplaces in Australia [17, 18].
- 1987, 1995 and 2006, the Australian Government introduced a series of new graphic health warnings on tobacco products [19-21].
- 1998-99, The introduction of the per stick excise duty to replace the existing weight-based system in November 1999 resulted in an increase in cigarette prices [22, 23].
- 2000, the GST was introduced in Australia and average price paid per cigarette was increased by 17.7%; smoke-free policy in public places introduced in 2000 [22, 24].
- 2005, Australia joined the WHO Framework Convention on Tobacco Control (FCTC) in February [25, 26].
- 2010, the excise and excise-equivalent customs duty rate was increased by 25% [27, 28].
- 2011-12, a ban or restriction of promotion and display in retail outlets was introduced in all states [29, 30].

***Key alcohol policies/events after 1950s***

- 1960s, Liquor licence liberalization, restaurant & bar licences introduced, and relaxed trading hours [5, 31] across most Australian states.
- 1967-1974, Lowered minimum drinking age in 4 out of 8 Australian states, including legal drinking age in Western Australia changed from 21 to 18 in 1970; drinking age in Tasmania decreased from 21 to 20 in 1967 and then dropped to 18 in 1974; drinking age in South Australia lowered from 21 to 20 in 1968 and then dropped to 18 in 1971; legal drinking age in Queensland decreased from 21 to 18 in 1974 [32, 33].
- 1976-82, Random Breath Testing program was first introduced in Victoria in 1976 and then implemented across all Australian states in 1982 [34, 35].
- 1985-86, the National Campaign against Drug Abuse launched in 1985 [36, 37], clear drinking guidelines set out in 1986 by National Health and Medical Research Council [38].
- 2007-2013, Australian state governments rewrote their alcohol legislation (the density of outlets, trading hours and license conditions) in the context of increasing rates of alcohol-related harm [39] (e.g. ACT Liquor Act 2010 [40], Liquor Control Reform Regulations 2009 in VIC [41], Liquor Commission Rules 2007 in WA [42]and NSW Liquor Act 2007 [43]). For example, Northen Territory placed a wholesale ban on alcohol in particular high-risk areas in 2008 (e.g., the town of Katherine) and the Queensland Government stopped granting licenses that extend the trade of alcohol beyond midnight or before 5 am, from 2009 until 2013 [44, 45].
- 2008 Alcopops, in April 2008 the Australian Federal Government imposed a 70% tax increase on ‘ready to drink’ alcoholic beverages (RTDs, pre-mixed drinks, or “alcopops”) from $AUD39.36 to $AUD66.67 per litre of alcohol bringing the tax on RTDs into line with the tax on spirits [46, 47].

**1.3 Description of cancer mortality data (ICD-10)**

The ICD codes and description of cancer mortality data used in the study were summarized in the table below. It worth noting that the changes in ICD codes for cancer dieases in the last 60 years may have some impact on the cancer death records in Australia. Dummy variables for ICD changes were included in initial models to assess the impact of changes in coding practices, but there were no significant effects on cancer mortality rates, so they were excluded from the final models.

In this study we selected cancer mortality data based on following criteria:

1. The cancer site has associations with both alcohol and tobacco consumption (as we analysed jointed effects of alcohol and tobacco policy on these cancer mortality rate).
2. The cancer site has been analysed in previous studies with causal links documented in the clinical, case-control or cohort studies.
3. Long-term cancer mortality data is available between 1950s and 2013.

Based on these criteria, we excluded some types of cancer in our analysis, including cervix uteri, stomach, endometrium and cancer of the lower urinary tract. However, these specific cancer mortality rates were included in the overall cancer mortality rate.

Table S1. Description of cancer mortality data used in this study

| Cancer site | ICD-10 | Time period | Source |
| --- | --- | --- | --- |
| All cancers | C00-97, B21 | 1950 – 2013 | WHO Cancer Mortality Database |
| Breast | C50 | 1950 – 2013 | WHO Cancer Mortality Database |
| Colon, rectum and anus | C18-21 | 1955 – 2013 | WHO Cancer Mortality Database |
| Lip, oral cavity and pharynx | C00-14 | 1950 – 2013 | WHO Cancer Mortality Database |
| Liver | C22 | 1968 – 2013 | AIHW Cancer Mortality Database |
| Lung (incl. trachea and bronchus) | C33-34 | 1950 – 2013 | WHO Cancer Mortality Database |

**1.4 Development of dummy variables for key public health policies or events**

Table S2. Development of dummy variables for alcohol policy or events during 1960 and 1980s

| Year | Liquor license liberlisation in 1960s | Lowering drinking age in four states in 1970s | Introducing RBT in Victoria state in 1976 and implemented in all states since 1982 | National Campaign against Drug Abuse in 1985 & set out drinking guidelines in 1986 | Event decription |
| --- | --- | --- | --- | --- | --- |
| 1950 | 1 | 1 | 0 | 0 |  |
| 1951 | 1 | 1 | 0 | 0 |  |
| … | 1 | 1 | 0 | 0 |  |
| 1960 | 0 | 1 | 0 | 0 | Liquor licence liberalization started from 1960, liquor licences were introduced to restaurant and bar, and trading hours were relaxed. |
| 1961 | 0 | 1 | 0 | 0 |  |
| … | 0 | 1 | 0 | 0 |  |
| 1967 | 0 | 0.98 | 0 | 0 | The minimum legal drinking age was lowered from 21 to 20 in Tasmania in 1967. |
| 1968 | 0 | 0.95 | 0 | 0 | The minimum legal drinking age was lowered from 21 to 20 in South Australia in 1968. |
| 1969 | 0 | 0.95 | 0 | 0 |  |
| 1970 | 0 | 0.85 | 0 | 0 | The minimum legal drinking age was lowered from 21 to 18 in Western Australia in 1970. |
| 1971 | 0 | 0.80 | 0 | 0 | The minimum legal drinking age was lowered from 20 to 18 in South Australia in 1971. |
| 1972 | 0 | 0.80 | 0 | 0 |  |
| … | 0 | 0.80 | 0 | 0 |  |
| 1974 | 0 | 0.60 | 0 | 0 | In 1974, the legal drinking age in Tasmania was lowered from 20 to 18 and in Queensland it was lowered from 21 to 18. |
| 1975 | 0 | 0.60 | 0 | 0 |  |
| 1976 | 0 | 0.60 | 0.25 | 0 | RBT was first introduced in Victoria state in 1976 |
| … | 0 | 0.60 |  | 0 |  |
| 1980 | 0 | 0.60 | 0.26 | 0 | RBT was introduced in Northern Territory 1980 |
| 1981 | 0 | 0.60 | 0.33 | 0 | RBT was introduced in South Australia state in 1981 |
| 1982 | 0 | 0.60 | 0.67 | 0 | RBT was introduced in New South Wales and Australian Capital Territory in 1982 |
| 1983 | 0 | 0.60 | 0.69 | 0 | RBT introduced in Tasmania state in 1983 |
| … | 0 | 0.60 | 0.69 | 0 |  |
| 1985 | 0 | 0.60 | 0.69 | 0.5 | Launched the National Campaign against Drug Abuse in 1985 |
| 1986 | 0 | 0.60 | 0.69 | 1 | Set out clear drinking guidelines in 1986 by National Health Medical Research Council. |
| … | 0 | 0.60 | 0.69 | 1 |  |
| 1988 | 0 | 0.60 | 1 | 1 | RBT was introduced in Western Australian and Queensland states, and fully implemented in all Australian states and territories in 1988 |
| … | 0 | 0.60 | 1 | 1 |  |
| 2013 | 0 | 0.60 | 1 | 1 |  |

Table S3. Development of dummy variables for tobacco policy or events during 1960 and 1980s

| **Year** | Joint events of release of RCOP report in 1962 & USSG report in 1964 and dangers of tobacco media campaigns in 1967 | Ban cigarette ads on TV & radio in 1976 | State "Quit" Smoking Campaigns 1984-88 | Ban tobacco ads in print media in Dec 1986 and in troduce smoke free at workplace and health warning label in 1987 | Event description |
| --- | --- | --- | --- | --- | --- |
| 1950 | **0** | **0** | **0** | **0** |  |
| 1951 | **0** | **0** | **0** | **0** |  |
| … | **0** | **0** | **0** | **0** |  |
| 1962 | **0.33** | **0** | **0** | **0** | 1962, release of the Royal College of Physicians (RCOP) report on smoking in relation to cancer of the lung and other diseases. |
| 1963 | **0.33** | **0** | **0** | **0** |  |
| 1964 | **0.66** | **0** | **0** | **0** | 1964, release of United States Surgeon General (USSG) report indicated that that smoking is "the single greatest cause of avoidable morbidity and mortality in the United States”. |
| … | **0.66** | **0** | **0** | **0** |  |
| 1967 | **1** | **0** | **0** | **0** | Dangers of tobacco media campaigns in 1967 |
| … | **1** | **0** | **0** | **0** |  |
| 1975 | **1** | **0** | **0** | **0** |  |
| 1976 | **1** | **1** | **0** | **0** | A total ban on tobacco & cigarette advertisements on TV & radio commenced in September 1976. |
| … | **1** | **1** | **0** | **0** |  |
| 1983 | **1** | **1** | **1** | **0** | Statewide "Quit" Smoking Campaigns started in 1983 |
| 1984 | **1** | **1** | **1** | **0** |  |
| … | **1** | **1** | **1** | **0** |  |
| 1988 | **1** | **1** | **1** | **0** | Statewide "Quit" Smoking Campaigns finished in 1988 |
| … | **1** | **1** | **0** | **0** |  |
| 1985 | **1** | **1** | **0** | **0** |  |
| 1986 | **1** | **1** | **0** | **0** |  |
| 1987 | **1** | **1** | **0** | **1** | Tobacco advertising was banned in all print media in December 1986 and the Australian Government introduced a graphic health warnings on tobacco products and prohibited smoking in all enclosed workplaces in 1987. |
| 1988 | **1** | **1** | **0** | **1** |  |
| … | **1** | **1** | **0** | **1** |  |
| 2013 | **1** | **1** | **0** | **1** |  |

**1.5 Test for stationarity**

Table S4. Unit root test for stationarity of time series

|  | Augmented Dickey-Fuller Test on level data | | Augmented Dickey-Fuller Test on first differenced data | |
| --- | --- | --- | --- | --- |
|  | T-statistics | P-value | T-statistics | P-value |
| Alcohol consumption per capita | -0.260 | 0.588 | 1.969 | 0.047 |
| Tobacco consumption per capita | 1.028 | 0.996 | -7.588 | 0.000 |
| Male overall cancer mortality | 0.202 | 0.998 | -12.438 | 0.000 |
| Female overall cancer mortality | -0.509 | 0.980 | -9.324 | 0.000 |
| Female breast cancer mortality | -0.485 | 0.982 | -11.255 | 0.000 |
| Male colon, rectum and anus cancer mortality | 0.119 | 0.997 | -9.919 | 0.000 |
| Female colon, rectum and anus cancer mortality | -1.230 | 0.894 | -8.379 | 0.000 |
| Male lip, oral cavity and pharynx cancer mortality | -0.622 | 0.973 | -6.691 | 0.000 |
| Female lip, oral cavity and pharynx cancer mortality | -0.683 | 0.969 | -8.941 | 0.000 |
| Male liver cancer mortality | 1.528 | 0.998 | 4.483 | 0.002 |
| Female liver cancer mortality | 0.068 | 0.958 | -8.281 | 0.000 |
| Male lung cancer mortality | -1.390 | 0.151 | -1.976 | 0.047 |
| Female lung cancer mortality | -0.356 | 0.987 | -9.107 | 0.000 |

**1.6 Estimates of ARIMA models on the effects of alcohol and tobacco policy on alcohol and tobacco consumption**

Table S5. The estimated effects of alcohol and tobacco policy on alcohol and tobacco consumption

|  | Alcohol consumption |  |
| --- | --- | --- |
|  | Coef. (95% CIs) | S.E. |
| Liquor license liberalisation in 1960s | 0.236 (0.124, 0.348)* | 0.112 |
| Lowering drinking age in 1970s | 0.029 (-0.117, 0.175) | 0.146 |
| RBT introduced in Victoria state in 1976 and implemented in all states since 1982 | -0.303 (-0.43, -0.176)* | 0.127 |
| National Campaign against Drug Abuse in 1985 & drinking guidelines set out in 1986 | -0.048 (-0.154, 0.058) | 0.106 |
|  | Tobacco consumption |  |
|  | Coef. (95% CIs) | S.E. |
| Release of RCOP report in 1962, USSG report in 1964, and health media campaigns about the dangers of tobacco in 1967 | -0.080 (-0.098, -0.062)*** | 0.018 |
| Ban cigarette ads on TV & radio in 1976 | -0.071 (-0.103, -0.039)*** | 0.032 |
| State "Quit" Smoking Campaigns 1984-88 | -0.028 (-0.071, 0.015) | 0.043 |
| Ban tobacco ads in print media in Dec 1986 and introduce smoke-free workplace rules and health warning labels in 1987 | -0.036 (-0.050, -0.022)* | 0.014 |

Note: S.E. is standard errors. * *p*<0.05, ** *p*<0.01, *** *p*<0.001

**1.7 Alcohol and tobacco consumption (Age 15+) and skin cancer mortality in Australia**

We selected male and female skin cancer mortality as the reference group for readers to compare with patterns in Figure 3. Figure A1 has shown that the variations of male and female skin cancer were not influenced by the trend in alcohol and tobacco consumption in Austtralia.

Figure S1 Per capita alcohol and tobacco consumption (Age 15+) and skin cancer mortality in Australia

**References**

1. Scollo M, Winstanley M. Tobacco in Australia: Facts and issues. In. Edited by Victoria CC, 4th Ed. edn. Melbourne: Cancer Council Victoria; 2015.

2. Berridge V. Drugs, alcohol, and the First World War. *The Lancet.* 2014, 384(9957):1840-1841.

3. Wipfli H, Samet JM. One hundred years in the making: The global tobacco epidemic. *Annual Review of Public Health.* 2016, 37(1):149-166.

4. BARDSLEY P, OLEKALNS N. Cigarette and Tobacco Consumption: Have Anti-Smoking Policies Made a Difference? 1999, 75(3):225-240.

5. Moodie R. A brief history of alcohol consumption in Australia. In: The Conversation. Melbourne, Australia; 2013.

6. Proctor RN. The history of the discovery of the cigarette–lung cancer link: evidentiary traditions, corporate denial, global toll. 2012, 21(2):87-91.

7. the Centers for Disease Control and Prevention (CDC). Achievements in Public Health, 1900-1999: Tobacco Use -- United States, 1900-1999. *Morbidity and Mortality Weekly Report.* 1999, 48(43):986-993.

8. International Agency for Research on Cancer. Alcohol Drinking. IARC Monographs on the Evaluation of Carcinogenic Risks to Humans. In: Food / Nahrung. vol. 44. Lyon: International Agency for Research on Cancer; 1988.

9. Parliament A. Alcohol taxation in Australia. In. Edited by Parliamentary Budget Office. Canberra: Australian Parliament; 2015.

10. Health TDo. Tobacco taxation - The history of tobacco excise arrangements in Australia since 1901. In. Cancerra; 2018.

11. Physicians RCo. Smoking and health - Summary of a report of the Royal College of Physicians of London on smoking in relation to cancer of the lung and other diseases. In. London; 1962.

12. Health SGsACoSa. The Reports of the Surgeon General - Smoking and Health. In. Edited by U.S. Department of Health E, and Welfare. Washington D.C.: U.S. Government Printing Office; 1964.

13. National Archives of Australia. Tobacco advertising ban in Australia – Fact sheet 252. In. Edited by National Archives of Australia. Canberra: National Office; 1976.

14. Pierce JP, Dwyer T, Chamberlain A, Burke N, Frape G, Chapman S. Evaluation of the Sydney “Quit. For Life” anti-smoking campaign: Part 1. Achievement of intermediate goals. 1986, 144(7):341-344.

15. Chapman S. The news on smoking: newspaper coverage of smoking and health in Australia, 1987-88. 1989, 79(10):1419-1421.

16. Victorian Department of Human Services. Abuse and harm - legal and practice definitions Date of Advice: 23 April 2007 Advice no: 1008 Protecting Victoria’s Children, Child Protection Practice Manual is an on line application available via the Department of Human Services intranet for Department of Human Services Child Protection practitioners and managers. . In. Melbourne, Australia: Victorian Department of Human Services

2007.

17. Chapman S, Wakefield M. Tobacco Control Advocacy in Australia: Reflections on 30 Years of Progress. 2001, 28(3):274-289.

18. Victorian Parliament. Tobacco Act 1987. In. Melbourne: Victorian Parliament,; 1987.

19. Chapman S, Carter SM. “Avoid health warnings on all tobacco products for just as long as we can”: a history of Australian tobacco industry efforts to avoid, delay and dilute health warnings on cigarettes. 2003, 12(suppl 3):iii13-iii22.

20. Miller CL, Hill DJ, Quester PG, Hiller JE. The impact of Australia’s new graphic cigarette packet warnings on smokers’ beliefs and attitudes. *Australasian Marketing Journal (AMJ).* 2011, 19(3):181-188.

21. Government A. Health warnings. In. Edited by Health TDo. Canberra: Australian Government - The Department of Public Health; 2018.

22. Government A. Tobacco taxation - Tobacco excise arrangements in Australia since 1901. In. Edited by Health TDo. Canberra: Australian Government - The Department of Public Health; 2018.

23. Scollo M, Borland R. Taxation reform as a component of tobacco control policy in Australia. In. Geneva; 2004.

24. Scollo M, Younie S, Wakefield M, Freeman J, Icasiano F. Impact of tobacco tax reforms on tobacco prices and tobacco use in Australia. 2003, 12(suppl 2):ii59-ii66.

25. Zhou SY, Liberman JD, Ricafort E. The impact of the WHO Framework Convention on Tobacco Control in defending legal challenges to tobacco control measures. 2019, 28(Suppl 2):s113-s118.

26. Government A. WHO Framework Convention on Tobacco Control. In. Edited by Health TDo. Canberra: Australian Government - The Department of Public Health; 2018.

27. Scollo M, Zacher M, Coomber K, Bayly M, Wakefield M. Changes in use of types of tobacco products by pack sizes and price segments, prices paid and consumption following the introduction of plain packaging in Australia. 2015, 24(Suppl 2):ii66-ii75.

28. Government A. Tobacco excise. In. Edited by Health TDo. Canberra: Australian Government - The Department of Public Health; 2018.

29. Li L, Borland R, Fong GT, Thrasher JF, Hammond D, Cummings KM. Impact of point-of-sale tobacco display bans: findings from the International Tobacco Control Four Country Survey. *Health Educ. Res.* 2013, 28(5):898-910.

30. Zacher M, Germain D, Durkin S, Hayes L, Scollo M, Wakefield M. A Store Cohort Study of Compliance With a Point-of-Sale Cigarette Display Ban in Melbourne, Australia. *Nicotine & Tobacco Research.* 2012, 15(2):444-449.

31. Parliament of Victoria. Liquor, temperance and legislation: The origins of six o'clock closing in Victoria during WWI In. Melbourne: Parliament of Victoria,; 2016.

32. Jiang H, Livingston M, Manton E. The effects of random breath testing and lowering the minimum legal drinking age on traffic fatalities in Australian states. *Inj. Prev.* 2015, 21(2):77-83.

33. Smith DI, Burvill PW. Effect on Traffic Safety of Lowering the Drinking Age in Three Australian States. 1986, 16(2):183-198.

34. Jiang H, Livingston M, Room R. Alcohol consumption and fatal injuries in Australia before and after major traffic safety initiatives: A time series analysis. *Alcoholism: Clinical and Experimental Research.* 2015, 39(1):175-183.

35. Henstridge J, Homel R, Mackay P. The Long-Term Effects of Random Breath Testing in Four Australian States: A Time Series Analysis. In. Canberra: Department of Transport and Regional Development; 1997.

36. McDonald D. Australia's National Campaign Against Drug Abuse. *Australian Drug and Alcohol Review.* 1987, 6(3):169-170.

37. Miller M, Hamilton M, Flaherty B. The Evaluation of Australia's National Campaign against Drug Abuse. 1992, 22(3):487-506.

38. Bowden JA, Delfabbro P, Room R, Miller CL, Wilson C. Alcohol consumption and NHMRC guidelines: has the message got out, are people conforming and are they aware that alcohol causes cancer? 2014, 38(1):66-72.

39. Trifonoff A, Andrew R, Steenson T, Nicholas R, Roche AM. Liquor licensing legislation in Australia: An overview. In. Adelaide, SA: National Centre for Education and Training on Addiction (NCETA); 2011.

40. Government ACT. ACT Liquor Act 2010 In. Canberra: Australian Capital Territory Government; 2010.

41. Victorian Parliament. Liquor Control Reform Regulations 2009. In. Edited by Victorian Parliament. Melbourne; 2009.

42. Western Austrlaian Government. Liquor Commission Rules 2007 In. Edited by Western Austrlaian Government. Perth; 2007.

43. Menéndez P, Tusell F, Weatherburn D. The effects of liquor licensing restriction on alcohol-related violence in NSW, 2008–13. 2015, 110(10):1574-1582.

44. Howard SJ, Gordon R, Jones SC. Australian alcohol policy 2001–2013 and implications for public health. *BMC Public Health.* 2014, 14(1):848.

45. Alcohol and Health in Australia; 2012.

46. Gale M, Muscatello DJ, Dinh M, Byrnes J, Shakeshaft A, Hayen A *et al*. Alcopops, taxation and harm: a segmented time series analysis of emergency department presentations. 2015, 15(1):468.

47. DORAN CM, DIGIUSTO E. Using taxes to curb drinking: A report card on the Australian government's alcopops tax. 2011, 30(6):677-680.
